# Whole-Exome Sequencing Identified a Novel Homozygous Frameshift Mutation of *HPS3* in a Consanguineous Family with Hermansky-Pudlak Syndrome

**DOI:** 10.1155/2021/4535349

**Published:** 2021-09-24

**Authors:** Zhao-Xia Wang, Yi-Hui Liu, Yi Dong, Ya-Li Li, Tie-Yu Tang, Liang-Liang Fan

**Affiliations:** ^1^Department of Neurology, Affiliated Hospital of Yangzhou University, Yangzhou, Jiangsu 225001, China; ^2^Departments of Reproductive Genetics, Hebei General Hospital, Shijiazhuang, 050051, China; ^3^Department of Cell Biology, The School of Life Sciences, Central South University, Changsha 410013, China

## Abstract

Hermansky-Pudlak syndrome (HPS) is a rare genetic disorder with an autosomal recessive inherited pattern. It is mainly characterized by deficiencies in lysosome-related organelles, such as melanosomes and platelet-dense granules, and leads to albinism, visual impairment, nystagmus, and bleeding diathesis. A small number of patients will present with granulomatous colitis or fatal pulmonary fibrosis. At present, mutations in ten known genetic loci (*HPS1–11*) have been identified to be the genetic cause of HPS. In this study, we enrolled a consanguineous family who presented with typical HPS phenotypes, such as albinism, visual impairment, nystagmus, and bleeding diathesis. Whole-exome sequencing and Sanger sequencing were applied to explore the genetic lesions of the patient. A novel homozygous frameshift mutation (NM_032383.5, c.1231dupG/p.Aps411GlyfsTer32) of *HPS3* was identified and cosegregated in the family members. Furthermore, real-time PCR confirmed that the mutation decreased the expression of *HPS3*, which has been identified as the disease-causing gene of HPS type 3. According to ACMG guidelines, the novel mutation, resulting in a premature stop codon at amino acid 442, is a pathogenic variant. In summary, we identified a novel mutation (NM_032383.5, c.1231dupG/p.Aps411GlyfsTer32) of *HPS3* in a family with HPS. Our study expanded the variant spectrum of the *HPS3* gene and contributed to genetic counseling and prenatal genetic diagnosis of the family.

## 1. Introduction

Hermansky-Pudlak syndrome (HPS) is a rare genetic disorder and is inherited in an autosomal recessive manner. It is mainly characterized by oculocutaneous albinism, bleeding diathesis, and mild ceroid lipofuscinosis [1]. A previous study demonstrated that both albinism and bleeding diatheses are associated with defects in the biogenesis of lysosomes and therefore affect two specialized lysosome-related organelles: melanosomes and platelet-dense granules [2, 3]. Melanosome defects can lead to abnormal pigmentation, including nystagmus, decreased visual acuity, and hypopigmentation of skin, hair, and irides, and platelet granule disorders will cause prolonged bleeding, which usually shows bruising, epistaxis, and gingival bleeding as early symptoms of HPS [4, 5]. Some HPS patients also develop pulmonary fibrosis, a potentially fatal lung disease, and granulomatous colitis; the pathogenesis and cellular bases of both of these diseases are poorly understood [6].

At present, mutations in eleven known genetic loci (*HPS1–11*) have been identified as genetic lesions of HPS with an autosomal recessive inherited pattern [7, 8]. The proteins encoded by *HPS1–11* can be divided into four multiprotein complexes called the biogenesis of lysosome-related organelle complex (BLOC-1, BLOC-2, and BLOC-3) and adapter protein (AP-3) [2, 9]. The *HPS3* gene encodes a subunit of BLOC-2 that contains a potential clathrin-binding motif, consensus dileucine signals, and tyrosine-based sorting signals for targeting vesicles of lysosomal lineage. *HPS3* is located on chromosome 3q24 and consists of 17 exons spanning ~113.7 kilobases [10]. To date, 39 mutations of *HPS3* have been reported to be associated with HPS type 3 across different populations. The HPS3 protein is also called BLOC2S1, and HPS3, HPS5, and HPS6 comprise the BLOC-2 protein. Previous studies have suggested that BLOC-2 can regulate the transportation of membrane proteins from endosomal domains to lysosome-related organelles, including melanosomes and platelet-dense granules. Malfunction of the HPS3 protein may affect the stability of BLOC-2, which may further affect membrane trafficking and lead to albinism and platelet dysfunction [11]. At present, studies have found that mutations in BLOC-2-related genes result in a relatively mild phenotype without life-threatening features [12].

In this study, we enrolled a consanguineous family presented with typical HPS phenotypes such as albinism, visual impairment, nystagmus, and bleeding diathesis. Whole-exome sequencing and Sanger sequencing suggested that a novel homozygous frameshift mutation (NM_032383.5, c.1231dupG/p.Aps411GlyfsTer32) of *HPS3* may be the genetic lesion of the family.

## 2. Materials and Methods

### 2.1. Subjects

**Figure 1:**
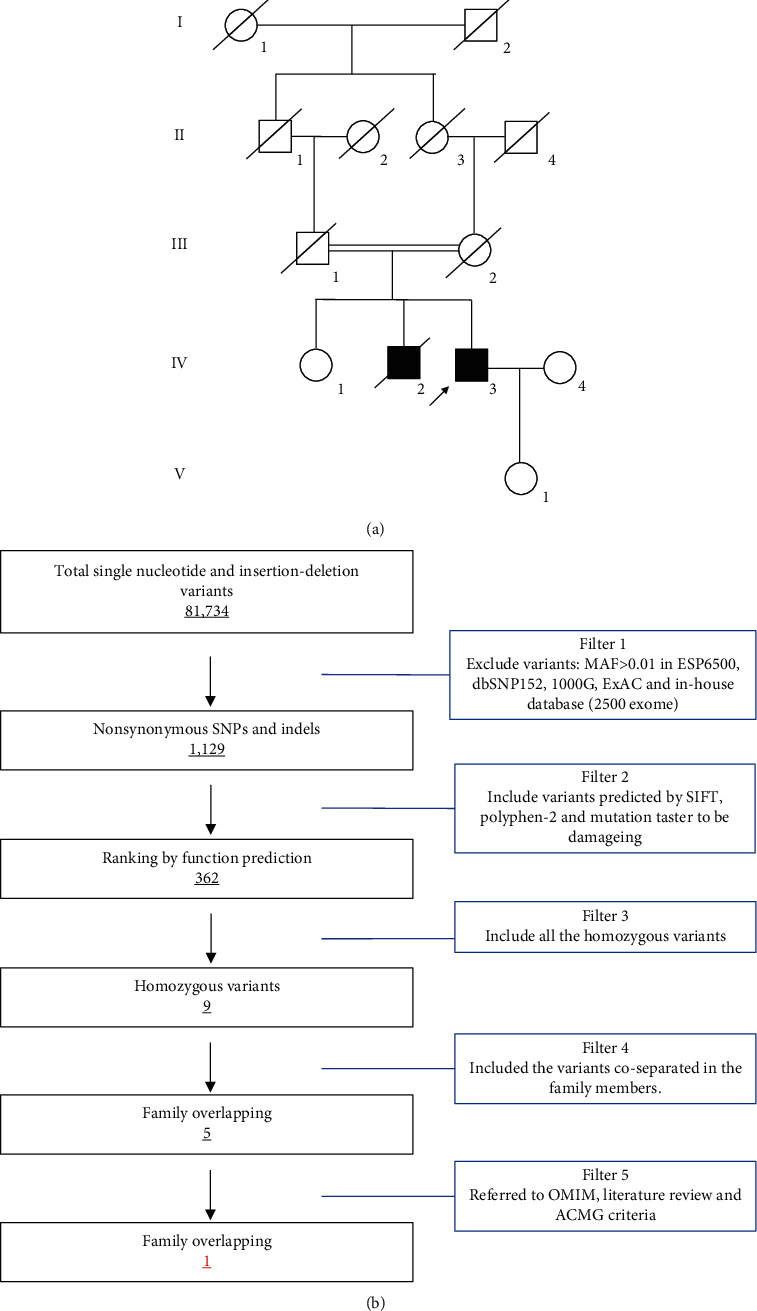
The pedigree and hair color of the proband. (a) Pedigree of the family. The pedigree shows five generations of the family. Roman numerals refer to generations. Circles refer to female subjects. Squares refer to male subjects. Solid symbols refer to affected subjects. Crossed-out symbols refer to deceased subjects. The arrow indicates the proband. (b) Schematic representation of the filter strategies employed in this study.

All 13 family members were investigated in this study (Figure 1(a)). The peripheral blood samples of one patient (IV-3) and three unaffected family members (IV-1, IV-4, and V-1) were collected. Clinical data, such as ophthalmologic examination, thromboelastogram platelet mapping assay, and platelet aggregation assay, were recorded carefully. The studies involving participants were reviewed and approved by the Ethics Committee of the Affiliated Hospital of Yangzhou University (Yangzhou, China). The patients/participants provided their written informed consent to participate in this study. Written informed consent was obtained from the individual(s) for the publication of any potentially identifiable images or data included in this study.

### 2.2. Whole-Exome Sequencing

The proband (IV-3) was subjected to whole-exome sequencing analysis. Genomic DNA was extracted from peripheral blood lymphocytes of all the family members with a DNeasy Blood & Tissue Kit (Qiagen, Valencia, CA) following the manufacturer's instruction. Exome capture and next-generation sequencing were provided by the BerryGenomic Institute (Beijing, China). One microgram of qualified genomic DNA from the patient was captured by the Agilent's SureSelect Human All Exon kit V5 (Agilent Technologies, Inc., Santa Clara, USA) and sequenced by Illumina Hiseq4000 (Illumina Inc., San Diego, USA). Shortly, genomic DNAs were randomly carved by Covaris S220 sonicator (Covaris, Inc., Woburn, USA) [13]. Then, the fragmented DNAs underwent three enzymatic steps: end repair, A-tailing, and adapter ligation. The adapter-ligated DNA fragments were amplified with Herculase II Fusion DNA Polymerase (Agilent). Finally, the exomes in the precapture libraries were captured by SureSelect capture library kit (Agilent) [14]. After DNA quality assessment, the captured DNA library was used for next-generation sequencing on the Illumina Hiseq4000 platform [13]. Downstream processing was carried out by Genome Analysis Toolkit (GATK), Varscan2, and Picard, and variant calls were made with the GATK Haplotype Caller [13]. Variant annotation referred to Ensemble release 82, and filtering was conducted by ANNOVAR Documentation [15–17].

The strategies of data filtering are as follows (Figure 1(b)): (a) nonsynonymous SNPs or frameshift-causing INDELs with an alternative allele frequency > 0.01 in the NHLBI Exome Sequencing Project Exome Variant Server (ESP6500), dbSNP152 (http://www.ncbi.nlm.nih.gov/projects/SNP/index.html), the 1000 Genomes project (http://www.1000genomes.org/), the ExAC database (http://exac.broadinstitute.org), or in-house exome databases of BerryGenomic (2000 exomes) were excluded; (b) the filtered SNPs and INDELs, predicted by SIFT (http://sift.jcvi.org/), Polyphen2 (http://genetics.bwh.harvard.edu/pph2/), and MutationTaster (http://www.mutationtaster.org/) that were damaging remained; (c) all the homozygous mutations remained; and (d) cosegregation analysis was conducted in the family mutations.

### 2.3. Mutation Validation and Cosegregation Analysis

Sanger sequencing was applied to validate the candidate variants identified in whole-exome sequencing. Cosegregation analysis was conducted in all family members of this study. The primer pairs were designed by Primer 5 (the primers were as follows: forward: 5′-TTGGTTCTTGGCCTCACTTT-3′ and reverse: 5′-GTTCTTGCCTAGCTTAGGTCTG-3′), and sequences of the polymerase chain reaction (PCR) products were determined using the ABI 3100 Genetic Analyzer (ABI, Foster City, CA).

### 2.4. RNA Extraction and Real-Time qPCR

Total RNA was extracted by the PureLink® RNA Mini Kit (Thermo Fisher Scientific, #12183025) from affected patient and healthy control skin biopsies. cDNA was synthesized from a total of 1 *μ*g of RNA using the RevertAid First Strand cDNA Synthesis Kit (Thermo Fisher Scientific, #K1621) with oligo (dT) primers. Real-time qPCR reactions were carried out in Fast 7500 Real-Time PCR Systems (Applied Biosystems) using Maxima SYBR Green/ROX qPCR Master Mix (2×) (Thermo Fisher Scientific, #K0221). The primers for real-time PCR were located in exon 1 and exon 2 of the *HPS3* gene: forward: 5′-CAGCGAGGCTGGAGATTATTT-3′ and reverse: 5′-CATTCGGATACACACACGAGAG-3′. The 2^(-△△Ct)^ method was used to compare HPS3 mRNA expression between the affected individuals and the controls. Each assay was performed in three independent tests. The data were analyzed by unpaired two-tailed tests using GraphPad Prism V.5 software (V.5.0).

## 3. Results

### 3.1. Clinical Data

**Figure 2:**
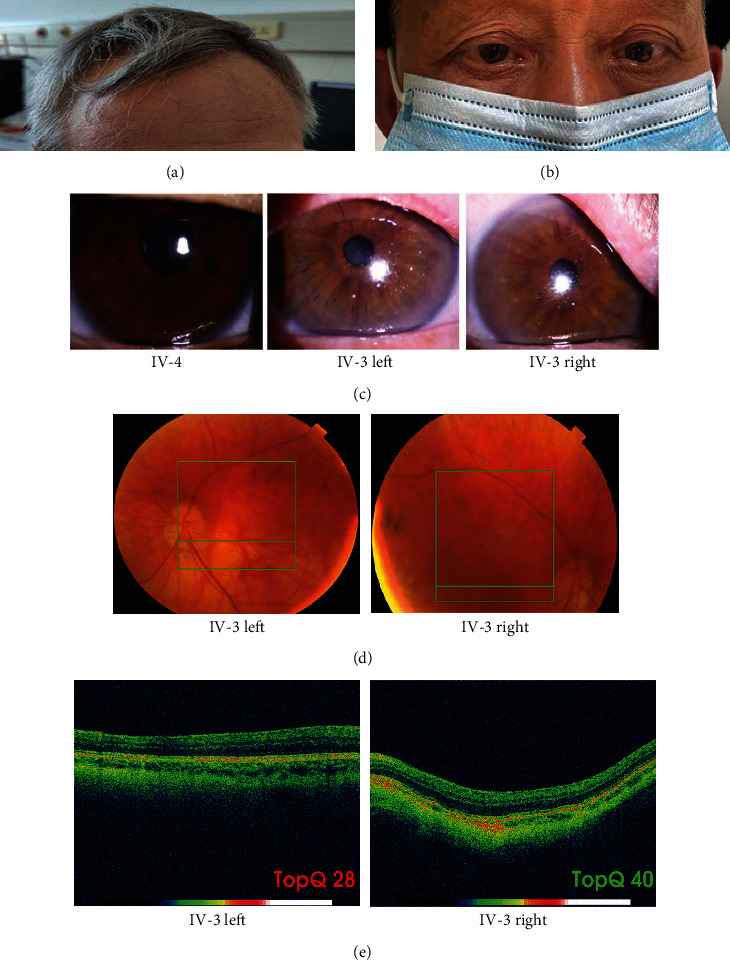
The ophthalmologic examination data of the proband. (a) Photograph showing the hair color of the proband. (b) Photograph showing the eye position of the proband. (c) Iris transillumination. Proband's iris with transillumination defects. (d) Fundus examination showing retinal pigmentation due to patchy areas lacking retinal pigment epithelium. (e) OCT examination showing locally thickened retinal epithelium in the macular area and disappearance of fovea structure.

The proband (IV-3), a 65-year-old man, presented with gray hair (Figure 2(a)), esotropia (Figure 2(b)), albinism of iris edges (Figure 2(c)), decreased visual acuity, congenital nystagmus, and hemorrhagic diathesis. A medical history survey revealed that the proband presented with gray hair, albinism of the iris, and nystagmus at eight years of age, and two years later, the vision of the proband decreased significantly. According to his daughter's (V-1) memories, the proband suffered from recurrent epistaxis and gum bleeding 20 years prior but did not accept formal treatment. Ophthalmologic examination showed decreased visual acuity (left 0.2 and right 0.3) and a patchy pattern caused by retinal pigmentation (Figure 2(d)). Optical coherence tomography (OCT) demonstrated locally thickened retinal epithelium in the macular area and disappearance of the foveal structure of both eyes (Figure 2(e)). Routine blood tests and routine coagulation function tests, including activated partial thromboplastin, prothrombin time, international normalized ratio, thrombin time, fibrinogen, and D-dimer, in the proband were normal. Thromboelastography platelet mapping assays (Table 1) and platelet aggregation assays (Table 2) found that the function of platelets was disrupted in the proband. The *K* value increased to 3.9 min, and the angle decreased to 43.7°. The AA_MA and ADP_MA both reached 14.3 mm and 14.7 mm, respectively, and the AA and ADP inhibition ratio increased to 78.8% and 78.1%, respectively. After EPI, Coll, and ACA induction, the ratio of platelet aggregation decreased to 48.2%, 7.2%, and 60.3%, respectively. Family history investigation revealed that the proband's mother and father were intermarried, and his brother (IV-2) also presented with nystagmus but died two years prior in a traffic accident.

### 3.2. Genetic Analysis

**Figure 3:**
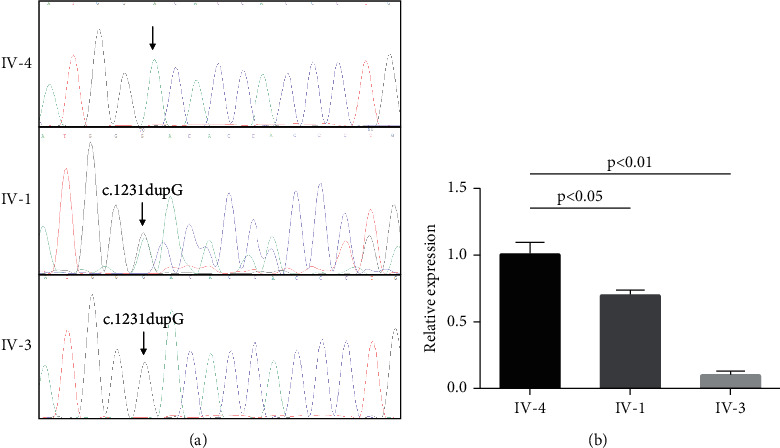
Genetic analysis of the family. (a) Sanger DNA sequencing chromatogram demonstrates the homozygosity for the mutation (c.1231dupG) of *HPS3* in the proband. (b) Real-time PCR detects the expression of HPS3 in the skin biopsy of IV-4, IV-1, and IV-3.

Whole-exome sequencing yielded 10.11 Gb of data with 99.7% coverage of the target region and 98.6% of the target covered over 10×. After alignment and single nucleotide variant calling, 81,734 variants were identified in the proband. Via the abovementioned filtering method and Sanger sequencing validation, five homozygous mutations (*SUPT20HL1*: c.2078C>T/p.Ser693Leu, *FAM47A*: c.1004C>T:p.Ser335Phe, *HMCN2*: c.4087A>T/p.Arg1363Trp, *KIR2DL4*: c.810dupG/p.Met271AsnfsTer141, and *HPS3*: c.1231dupG/p.Aps411GlyfsTer32) were identified in the proband. Among these five homozygous mutations, only the frameshift mutation (NM_032383.5, c.1231dupG/p.D411GfsX32) of *HPS3* was associated with the proband's phenotypes (Figure 3(a)). No other potential pathogenic mutations for HPS-related disease phenotypes were found. The novel mutation, generating a truncated protein in exon 6 of the *HSP3* gene, was absent in our 200 local control participants. Bioinformatics programs predicted that this mutation (NM_032383.5, c.1231dupG/p.Aps411GlyfsTer32) was a pathogenic mutation and was located in an evolutionarily conserved site of the HPS3 protein. According to ACMG guidelines [18], this mutation is pathogenic (PVS1+PM2+PM3+PP1+PP3+PP4). We then extracted the total RNA from a skin biopsy from the proband and other family members. After reverse transcription PCR, real-time PCR detection found that, compared with the healthy controls, the mRNA level of HPS3 was decreased by approximately 30% in the heterozygous carrier and 90% in the homozygous mutation carrier (Figure 3(b)). These results suggest that this variant (NM_032383.5, c.1231dupG/p.Aps411GlyfsTer32) of *HSP3* is a loss-of-function mutation and can lead to nonsense-mediated mRNA decay.

## 4. Discussion

HPS is a rare autosomal recessive disorder first described by Hermansky and Pudlak in 1959 [19]. The most common symptoms of HPS are hypopigmentation, loss of visual acuity, nystagmus, bleeding diathesis, and, in some cases, granulomatous colitis and fatal pulmonary fibrosis [7, 9]. In this study, we identified a novel homozygous frameshift mutation (NM_032383.5, c.1231dupG/p.Aps411GlyfsTer32) of *HPS3* in a consanguineous family that presented with typical HPS phenotypes such as albinism, visual impairment, nystagmus, pulmonary fibrosis, and bleeding diathesis. Our study confirmed the clinical diagnosis and further aided in the diagnosis of HPS type 3 in the proband.

A previous study proved that the HPS3 protein contained two main functional regions: a clathrin-binding motif (172 aa-176 aa) and an endoplasmic reticulum (ER) retention signal (1000 aa-1003 aa) [20]. Previous studies have proven that the clathrin-binding motif helps HPS3 associate with clathrin, predominantly on small clathrin-containing vesicles in the perinuclear region [20]. The ER retention signal was responsible for vesicle trafficking from the ER and Golgi [21, 22]. In this study, this mutation (NM_032383.5, c.1231dupG/p.Aps411GlyfsTer32) of *HPS3* may affect the expression of *HPS3* and then affect the formation of the BLOC-2 protein complex. Thus, it ultimately disrupts the formation and trafficking of vesicles and leads to the lysosome-related disease HPS type 3.

Previous studies have reported that different types of HPS patients showed various degrees of visual impairment. Visual impairment is usually less severe in patients with HPS type 3 than in those with HPS type 1 [23]. In the present study, the proband with HPS3 showed a very mild decrease in visual acuity, so these findings correspond to previous studies. Meanwhile, our study showed similar results in iris transillumination examination [23]. As previously reported, patients with HPS type 3 had less iris transillumination than patients with HPS type 1 [24]. Indeed, in our study, the proband with HPS type 3 had minimal iris transillumination.

Strabismus is another common characteristic in HPS patients [23]. A previous report showed that different types of strabismus may be associated with various types of albinism in HPS patients [25]. Exotropia was more frequent in HPS type 3 patients, but in our study, the HPS type 3 patient showed very slight left eye esotropia, which is typically more common in HPS type 1 patients. This indicates that the genotype-phenotype correlations in HPS variants require more clinical data and further research.

Bleeding diathesis is also an important symptom of HPS3 that is mainly caused by defects in the platelet dense body [12]. Platelet dense bodies contain calcium, polyphosphates, serotonin (5-HT), adenosine triphosphate (ATP), and adenosine diphosphate (ADP). Since dense granules release their contents when stimulated and ADP can further induce aggregation of other platelets, defects in platelet dense bodies will cause storage pool deficiency and therefore coagulation dysfunction [26]. In our study, the patient presented with bleeding diathesis since his childhood, and it became less severe over time. In the platelet aggregation assay, platelet aggregation ratios correspondingly decreased when induced by EPI, Coll, and ACA. This was especially true in Coll induction, where the platelet aggregation ratio reached 7.2%. In the TEG platelet mapping test, the patient still showed a slight bleeding tendency, even though he was not taking any antiplatelet drugs.

Some HPS patients may have ceroid deposition resulting in pulmonary fibrosis or simultaneous granulomatous colitis [6]. However, no phenotype of inflammatory bowel disease or pulmonary fibrosis in *HPS3* mutations has been reported in the literature. The proband in our study did not present with any symptoms of the respiratory or digestive systems. The patient's chest CT only indicated mild asymptomatic bronchiectasis, and the lung function test was also normal. These results were consistent with the findings in previous studies.

## 5. Conclusions

In summary, the present study identified a novel *HPS3* homozygous mutation (c.1231dupG/p.Aps411GlyfsTer32) in a consanguineous family presented with typical HPS phenotypes including albinism, visual impairment, nystagmus, and bleeding diathesis. The results of the present study offer further support for the significant involvement of *HPS3* in HPS. The results also expand on the spectrum of *HPS3* mutations and contribute to the genetic diagnosis and counseling of families with HPS type 3.

## Figures and Tables

**Table 1:** TEG platelet mapping assay for the family members.

Family member	*R* (min)	*K* (min)	Angle (°)	MA (mm)	*G* (d/sc)	EPL (%)	LY30 (%)	A_MA (mm)	AA_MA (mm)	AA (%)	ADP_MA (mm)	ADP (%)
IV-3	8.4	3.9	43.7	57.5	6764	0.1	0.1	2.7	14.3	78.8	14.7	78.1
IV-1	6.5	2.1	61.1	67.3	10278	0.1	0.4	6.1	38	47.9	40.8	43.3
IV-4	5.5	2.1	65.1	62.9	8465	0.1	0.3	4.5	38.6	41.6	27.7	60.3
V-1	7.8	1.9	58.8	57.5	6758	0.1	0.1	9.3	34.3	48.1	36.6	43.4

AA: arachidonic acid; ADP: adenosine diphosphate.

**Table 2:** Platelet aggregation assay for the family members.

Family member	ADP (%)	EPI (%)	Coll (%)	ACA (%)
IV-3	82.6	48.2	7.2	60.3
IV-1	74.4	80.3	78.1	80.1
IV-4	70.6	66.6	85.6	83.8
V-1	84.4	78.4	79.9	74.4

ADP: adenosine diphosphate; EPI: epinephrine; Coll: collagen; ACA: arachidonic acid.

## Data Availability

The data used to support the findings of this study are available from the corresponding author upon request.
